# The fragile X syndrome–autism comorbidity: what do we really know?

**DOI:** 10.3389/fgene.2014.00355

**Published:** 2014-10-16

**Authors:** Leonard Abbeduto, Andrea McDuffie, Angela John Thurman

**Affiliations:** ^1^MIND Institute, University of California, Davis, Sacramento, CA, USA; ^2^Department of Psychiatry and Behavioral Sciences, University of California, Davis, Sacramento, CA, USA

**Keywords:** fragile X syndrome, autism spectrum disorder, comorbid conditions, language and communication impairments, psychiatric conditions

## Abstract

Autism spectrum disorder (ASD) is a common comorbid condition in people with fragile X syndrome (FXS). It has been assumed that ASD symptoms reflect the same underlying psychological and neurobiological impairments in both FXS and non-syndromic ASD, which has led to the claim that targeted pharmaceutical treatments that are efficacious for core symptoms of FXS are likely to be beneficial for non-syndromic ASD as well. In contrast, we present evidence from a variety of sources suggesting that there are important differences in ASD symptoms, behavioral and psychiatric correlates, and developmental trajectories between individuals with comorbid FXS and ASD and those with non-syndromic ASD. We also present evidence suggesting that social impairments may not distinguish individuals with FXS with and without ASD. Finally, we present data that demonstrate that the neurobiological substrates of the behavioral impairments, including those reflecting core ASD symptoms, are different in FXS and non-syndromic ASD. Together, these data suggest that there are clinically important differences between FXS and non-syndromic ASD that are masked by reliance on the categorical diagnosis of ASD. We argue for use of a symptom-based approach in future research, including studies designed to evaluate treatment efficacy.

## INTRODUCTION

Autism spectrum disorder (ASD) is a common comorbid condition in people with fragile X syndrome (FXS). The prevalence of ASD in FXS has been estimated at 50%, although there is considerable variability across studies (Demark et al., 2003; Kaufmann et al., 2004; Harris et al., 2005; Budimirovic and Kaufmann, 2011). This high rate of co-occurrence has led many researchers to suggest that FXS, being an etiologically “simpler” (i.e., a single-gene) disorder, will provide insights into the etiology of non-syndromic ASD (Belmonte and Bourgeron, 2006). Arguably, these insights have yet to materialize. In part, this reflects the fact that although the comorbidity of FXS and ASD was first documented more than 30 years ago (Brown et al., 1982), there is still much that we do not understand about the comorbidity. Most importantly, we contend that it is not clear that the ASD diagnosis in FXS reflects the same underlying psychological (and thus, neurobiological) impairments as in non-syndromic ASD. In other words, we lack a definitive answer to the question, “Is ASD in FXS ‘true’ ASD?” and the current evidence is pointing to the possibility of a negative answer. Answering this question is critically important, especially at this time, because of recent claims that targeted pharmaceutical treatments found to be efficacious for core symptoms of FXS are likely to be beneficial for individuals with non-syndromic ASD as well (Berry-Kravis et al., 2012; Gurkan and Hagerman, 2012; Hagerman et al., 2012). To the extent that an ASD diagnosis reflects different or even partly different underlying impairments in FXS and non-syndromic cases, the possibility of shared treatments is reduced as well. In this paper, we present evidence suggesting that ASD in FXS and non-syndromic ASD may differ in important ways.

In trying to understand the FXS–ASD comorbidity, we have turned to five types of data. First, we have explored the individual symptoms leading to the diagnosis of ASD in FXS and the non-syndromic case; in essence, searching for evidence of similarities and differences in the symptom profile “earning” affected individuals the diagnosis. Second, we have examined behaviors and impairments that, although not part of ASD diagnostic criteria, are correlated with the diagnosis or correlated with autism symptom severity, again looking for differences and similarities between FXS and non-syndromic ASD. Third, we have explored the developmental trajectory of autism symptoms in FXS, comparing it to that in non-syndromic ASD, to discern similarities and differences between the two conditions. Fourth, we have sought to understand the symptoms and correlates distinguishing individuals with FXS who receive an ASD diagnosis from those with FXS who do not receive the diagnosis in an attempt to understand the nature of the difference between the two groups of individuals. Fifth, we have begun to consider the neurobiological underpinnings of ASD symptoms in FXS and in relation to the non-syndromic case.

In this article, we focus largely on our own research; however, we integrate other published literature to illustrate points of convergence and divergence from our own data. Note, however, that the review is not exhaustive; instead, we are selective, with the aim being to illustrate the pitfalls of assuming the equivalence of a categorical ASD diagnosis in FXS and the non-sndromic case (Harris, 2011). We conclude by offering an alternative conceptualization of the FXS–ASD comorbidity and of the nature of the insights each disorder can provide with regard to understanding the other. Useful reviews of the comorbid conditions associated with FXS, including ASD, can be found in Belmonte and Bourgeron (2006), Budimirovic and Kaufmann (2011), and Kaufmann et al. (2008).

## METHODOLOGICAL CONCERN: CONFOUNDING OF ASD AND IQ

In any examination of the comorbidity of FXS and ASD, one must address the inherent confound of cognitive ability (e.g., IQ) with ASD symptomatology in FXS. In FXS, individuals with a comorbid ASD diagnosis have lower IQs, on average, than those without the diagnosis and this characteristic is present over the life course (Bailey et al., 2001, 2000, 2001; Rogers et al., 2001; Kau et al., 2004; Kaufmann et al., 2004; Philofsky et al., 2004; Lewis et al., 2006; Hernandez et al., 2009; McDuffie et al., 2010). Quantitative metrics of autism symptom severity also are related to IQ in individuals with FXS (e.g., Hatton et al., 2006), although this relationship is largely accounted for by the influence of fragile X mental retardation protein (FMRP) on both variables (Kover et al., 2013). In non-syndromic ASD, the concurrent relationship between IQ and autism symptoms is not linear (Charman et al., 2011; Wang et al., 2013).

The fact that the relationship between cognitive ability and ASD symptomatology differs for FXS and non-syndromic ASD complicates any comparison between the two disorders because, on average, cognitive abilities are more limited in individuals with FXS than in individuals with non-syndromic ASD; for example, more than 90% of males with FXS have IQs in the range of intellectual disability (Hessl et al., 2009) compared to 16–38% of males with non-syndromic ASD (Baio, 2012; Ryland et al., 2014). Unless differences in cognitive ability are controlled in comparisons between FXS and non-syndromic ASD or between individuals with FXS with and without comorbid ASD, it is impossible to unambiguously ascribe differences in ASD symptom profiles, correlates, or developmental trajectories to etiological group or to cognitive ability. In the subsequent sections, the data reported are based on comparisons controlling for cognitive differences unless otherwise noted.

As a final point, it is worth noting that comparisons of FXS and non-syndromic ASD that involve IQ-matched samples have the disadvantage of truncating the IQ range for non-syndromic ASD and thereby limit generalizability of the findings. Put differently, largely only individuals with non-syndromic ASD and an intellectual disability will be included in such matched comparisons despite the fact that they represent less than half of cases of non-syndromic IQ (Baio, 2012; Ryland et al., 2014). This limitation can be overcome in part by studying individuals with the *Fragile X Mental Retardation 1 (FMR1)* premutation, who can exhibit autism symptoms while displaying average-range IQs. In the present paper, however, we focus on individuals with the FXS full mutation because there has been substantially more research on the ASD comorbidity in FXS than in the premutation case. Moreover, the prevalence of ASD is higher in individuals with FXS than in the premutation case (Bailey et al., 2008), making the clinical importance of the issues more acute for FXS. For reviews of the literature on ASD and premutation carriers, see Losh et al. (2012) and Wheeler et al. (2014).

## METHODOLOGICAL CONCERN: MEASURE LIMITATIONS

The conclusions reached about ASD in FXS will be intimately tied to the measures used to assess ASD symptoms and status. Three cautions must be raised in this regard. First, different measures of ASD have been used across studies, with some measures emphasizing retrospective informant report and others observation of current behavior. The latter measures can differ in the standardization of the conditions of observation, from specific prompts to concatenations of impressions across naturally occurring activities. Such variability can lead to discrepant results across studies. Second, many of the measures available have been created for, and normed on, non-syndromic ASD cases, leaving their appropriateness for characterizing FXS uncertain. More importantly, these measures may lead to a “forced” fit between the impairments presented by the individual with FXS and the scoring rubrics, with the result being a “loss” of clinically important nuances in symptoms. Third, in some studies, a decision about whether a participant “has ASD” is based on meeting specified criteria on one or more standardized measure, whereas in other studies clinical judgment plays a role along with meeting criteria on standardized measures. This difference in decision making is akin to a difference between classification and true clinical diagnosis and can also lead to discrepant results across studies. In the present article, we do not emphasize these issues in our study by study review for the sake of conciseness and because our goal is to provide an illustration of the complex and clinically important nuances lost by a failure to move beyond a simple ASD or no ASD categorization of individuals with FXS.

## ASD SYMPTOM PROFILES IN FXS AND NON-SYNDROMIC CASES

Perhaps the most direct approach to addressing the question of whether ASD in FXS reflects the same underlying psychological impairments as in non-syndromic ASD is to compare the behaviors that lead to the diagnosis in the two conditions. Presumably, if ASD in FXS is “true” ASD, the symptom profiles leading to the diagnosis should be indistinguishable in the two conditions. In a recent study (McDuffie et al., 2014), we compared the ASD symptom profiles of 4- to 10-year-old boys with FXS who met criteria for ASD (*n* = 40) to chronological age-matched boys with ASD for whom FXS had been ruled out (*n* = 39), with the ASD diagnostic classification determined by the combined use of the Autism Diagnostic Observation Schedule (ADOS; Lord et al., 2007) and Autism Diagnostic Interview-Revised (ADI-R; Rutter et al., 2003) using the procedures described by Risi et al. (2006). Because, as noted, FXS is almost invariably associated with below average IQ in males, the sample was restricted at the outset to boys whose non-verbal IQs were under 85 (i.e., one standard deviation below the mean for the test) on the Leiter International Performance Scale-Revised (Roid and Miller, 1997). Symptom profiles were examined using the scores assigned for the child’s current functioning on 28 items of the ADI-R [10 in the Reciprocal Social Interaction (RSI) domain, 10 in the Communication (Comm) domain, and 8 in the Restricted Interests and Stereotyped Behaviors (RBS) domain].

In general, the boys with FXS and comorbid ASD showed less severe symptoms (i.e., lower scores) than did the boys with nonsyndromic ASD; however, significant differences emerged only for two items in the RSI domain (social smiling and showing/directing attention), one item in the Comm domain (pronominal reversal), and two items in the RBS domain (unusual preoccupations and compulsions/rituals). Thus, despite earning an ASD diagnosis, young boys with FXS are more socially responsive, less likely to engage in an atypical communicative behavior thought to be a “signature” of ASD (i.e., pronoun reversal), and less apt to display some higher-order repetitive behaviors than boys with non-syndromic ASD. Interestingly, these differences emerged despite the fact that, as would be expected in an age-matched comparison, the participants with FXS had lower IQs, on average, than did the boys with non-syndromic ASD, although, as noted, all had IQs less than 85.

In an additional analysis, McDuffie et al. (2014) selected a subset of the participants so as to create a FXS group and a non-syndromic ASD group (*n* = 21 per group) that not only met diagnostic criteria for ASD but also were matched on the overall severity of their ASD symptoms, using calibrated severity scores from the ADOS (Gotham et al., 2009). Even in this analysis, different symptom profiles emerged. As a group, the participants with FXS were significantly less impaired in terms of social smiling. There also were group differences that approached statistical significance in the range of facial expressions used to communicate (RSI), offering to share (RSI), the use of conventional/instrumental gestures (Comm), and unusual preoccupations (RBS), all involving less severe symptoms in participants with FXS. At the same time, however, the participants with FXS earned significantly higher (i.e., more impaired) scores on the display of complex mannerisms (RBS) and marginally higher scores on circumscribed interests (RBS). These results, perhaps even more dramatically than those of the previously described analysis comparing groups matched only on ASD diagnoses, suggest that the profiles of impairments that constitute ASD are partly different in FXS and non-syndromic ASD. Note that despite being matched on autism symptom severity, the non-verbal IQs of the boys with FXS were lower, although not significantly so, than those of the boys with non-syndromic ASD.

Matching on IQ, however, does not change the picture that emerged from the McDuffie et al.’s (2014) study. In a study of boys with minimal or no spoken language, Wolff et al. (2012) compared young boys with FXS and comorbid ASD to age- and IQ-matched boys with non-syndromic ASD on ADOS items in the RSI domain classified as measuring either social initiation or social response. These investigators found that the boys with FXS were less impaired than the boys with non-syndromic ASD in terms of social smiling, range of facial expressions used for communication, and response to joint attention, all suggesting less impairment in social responsiveness among those with FXS despite having an ASD diagnosis.

Although few studies have directly compared the ASD symptom profiles of individuals with FXS and comorbid ASD to those with non-syndromic ASD, several within-groups studies of FXS have suggested many important similarities to published findings on non-syndromic ASD, including social withdrawal, more severe receptive than expressive language deficits, and gaze avoidance (Budimirovic and Kaufmann, 2011). Moreover, it is important to note that even in the McDuffie et al.’s (2014) and Wolff et al.’s (2012) studies, the FXS and non-syndromic ASD groups were not significantly different on all, or even most, diagnostic symptoms.

Taken together, the studies to date demonstrate symptom overlap between FXS and non-syndromic ASD. At the same time, however, it is clear that important differences between the two groups are masked by assigning a categorical diagnosis of ASD. These symptom differences seem to suggest different underlying problems; for example, the social impairments in FXS may be less reflective, on average, of social indifference or a diminished appetite for social interaction.

## BEHAVIORAL CORRELATES OF ASD IN FXS AND NON-SYNDROMIC CASES

A second approach to addressing the question of whether ASD in FXS reflects the same underlying psychological impairments as in non-syndromic ASD is to examine the behavioral correlates of the ASD diagnosis and autism symptom severity. By examining behavioral correlates that are not part of the ASD diagnosis itself, one can begin to learn about the factors that influence and are influenced by ASD symptoms. To the extent that the pattern of correlations differs for FXS and non-syndromic ASD, the possibility of different underlying psychological impairments in the two conditions is increased. Several recent studies we have conducted taking this approach support the hypothesis that ASD in FXS differs in important ways from ASD in the non-syndromic case.

Thurman et al. (2014) used the Anxiety, Depression, and Mood Scale (ADAMS; Esbensen et al., 2003) to examine the co-occurrence of psychiatric symptomatology in 4- to 10-year-old boys with FXS or non-syndromic ASD. The ADAMS is an informant report measure standardized on a large sample of individuals with intellectual disabilities, which in the Thurman et al.’s (2014) study was completed by the children’s biological mothers. The ADAMS yields five subscale scores: Manic/Hyperactive Behavior; Depressed Mood; Social Avoidance; General Anxiety; and Obsessive/Compulsive Behavior. The investigators compared the groups using several different matching strategies involving various subgroups of the sample of 82 participants. The comparison of interest in the present context was one made between groups (*n* = 16 per group) matched on chronological age, IQ (assessed with the Leiter), and ASD symptom severity on the ADOS using the metric introduced by Gotham et al. (2009).

Three findings of interest emerged. First, boys with FXS received higher mean scores, reflective of greater psychiatric problems, on Manic/Hyperactive Behavior and General Anxiety than did the boys with non-syndromic ASD. Interestingly, these same scales were higher for the boys with FXS in the analyses involving other matching strategies, indicating their ubiquity over the full range of the FXS phenotypes. Second, the scales of the ADAMS were, with few exceptions, highly inter-correlated for the age-, IQ-, and ASD symptom severity-matched boys with FXS, with the strongest correlations involving the General Anxiety scale. In contrast, for the matched boys with non-syndromic ASD, the significant inter-correlations were far more sparse, with the only significant correlation being that between Social Avoidance and Obsessive/Compulsive Behavior. Third, the correlation between General Anxiety and Social Avoidance was significantly stronger in the FXS group than in the age-, IQ-, and severity-matched non-syndromic ASD group. Other researchers also have found links between anxiety and behaviors reflective of social avoidance in FXS (Kaufmann et al., 2008; Budimirovic and Kaufmann, 2011).

It is interesting to note first that there were substantial differences between the FXS and non-syndromic ASD groups as regards psychiatric findings despite their equivalence in ASD symptom severity in the Thurman et al.’s (2014) study. Thurman et al. (2014) argue that the preponderance of inattentive, hyperactive, and anxious behaviors in individuals with FXS may actually interfere with participation in, and the ability to learn from, social interaction, with these psychiatric factors thereby playing a cumulative role over time in the emergence of the social impairments that lead to an ASD diagnosis. Presumably, other factors, not measured in this study, are at play in the non-syndromic ASD case. Thurman et al. (2014) also suggest that the patterns of inter-correlations among subscales indicate that the symptoms queried on the ADAMS may tap different underlying psychological problems in FXS and non-syndromic ASD. In that vein, they argue that anxiety appears to be heightened and at the core of social avoidance in FXS, but not in non-syndromic ASD. Although anxiety is also a common comorbidity in non-syndromic ASD (Budimirovic and Kaufmann, 2011), other researchers also have found anxiety to be more problematic in FXS than in non-syndromic ASD (Cordeiro et al., 2011).

Other studies also have uncovered different patterns of correlations between autism symptoms and other behavioral problems in FXS and non-syndromic ASD. For example, Klusek et al. (2013) found that communication impairments were related to measures of vagal tone, which are thought to reflect physiological arousal and arousal regulation, for boys with non-syndromic ASD but not for boys with comorbid FXS and ASD. Again, such findings raise the possibility of different mechanisms underlying the same ASD symptoms in the two disorders.

Of course, there also are data suggesting similar patterns of correlation in FXS and non-syndromic ASD for some domains. Roberts et al. (2009), for example, found that baseline cortisol levels and change in cortisol during a stressor were correlated with social approach behavior for individuals with FXS and comorbid ASD as well as for individuals with non-syndromic ASD, but not for individuals with only FXS. Nevertheless, the fact that, as discussed in this section, there also are important differences in the patterns of correlations among behavioral domains in FXS and non-syndromic ASD, is consistent with the notion that at least partly different psychological and neurobiological mechanisms underlie the ASD diagnosis in the two conditions. Again, then, clinically important differences are obscured by reliance solely on the categorical diagnosis of ASD.

## DEVELOPMENTAL TRAJECTORY OF ASD SYMPTOMS IN FXS AND NON-SYNDROMIC CASES

Additional insight into the nature of the psychological impairments underlying ASD in FXS and non-syndromic ASD can be gleaned by studying the developmental course of ASD symptoms in the two conditions. More generally, Karmiloff-Smith (2012) has argued that similar phenotypes often belie important mechanistic differences that can only be discovered through studying the emergence of the phenotypes over the life course. In one stunning example, Karmiloff-Smith et al. (2003) have demonstrated that in infants with Williams syndrome numerosity skills are a relative strength and language a relative weaknesses, whereas in adults with Williams syndrome numerosity skills are relatively weak and language skills relatively strong.

In this vein, McDuffie et al. (2010) used retrospective data to track age-related changes in ASD symptoms in adolescents with FXS. In particular, these investigators conducted an in-depth analysis of ADI-R interviews completed by the biological mothers of 50 children and adolescents with FXS (both males and females), comparing the responses for those who met and did not meet criteria for autism. Assignment to the comorbid autism and no autism groups was determined following standard ADI-criteria for verbal individuals (i.e., participants were classified as having FXS + ASD if they met the designated lifetime cutoff scores for all ADI-R domains including age of onset). Change over the life course was examined by comparing maternal responses to the 29 ADI-R items that are queried both for current functioning and for the lifetime, or diagnostic, algorithm, with the latter items generally being anchored to functioning between the ages of 4 and 5 years.

Considerable change was observed in severity of autism symptoms from the diagnostic reference age to the current age in the McDuffie et al. (2010) sample, which had a mean chronological age near 13 years. In the comorbid ASD group, significant improvement was seen on 7 of 11 items in the RSI domain and 6 of 10 items in the Comm domain. In contrast, change was less evident in the Restricted Interests and Repetitive Behaviors domain, with only two of eight items showing significant improvement. In the case of the non-ASD participants with FXS, there was considerably less change from the diagnostic reference age of 4–5 years to the current ratings, which is not surprising given the relatively low (unimpaired) ADI-R scores assigned at the diagnostic reference age. As a result, at the current age of assessment, there was an attenuation of the difference in ASD symptoms between the two FXS groups with age.

Hernandez et al. (2009) also observed improvement of autism symptoms in their prospectively followed cohort of young boys with FXS, as well as a diminishing of differences between the groups with and without comorbid ASD. Unlike, McDuffie et al., however, these researchers found a worsening of autism symptoms in the FXS boys who did not initially meet criteria for an ASD diagnosis. The younger ages of the Hernandez et al.’s (2009) sample (i.e., 3–8 years) might account for the differing results across studies.

Unfortunately, neither McDuffie et al. (2010) nor Hernandez et al. (2009) included a non-syndromic ASD comparison group; however, comparisons of their findings with those of other studies focused on non-syndromic ASD are possible. In particular, like McDuffie et al. (2010); Shattuck et al. (2007) examined differences in ADI-R lifetime and current scores for a large group of children, adolescents, and young adults with non-syndromic ASD. In the Shattuck et al.’s (2007) sample, improvement with age was seen in all three domains of ASD symptomatology, a trend observed in several other studies as well (e.g., Piven et al., 1996; Gilchrist et al., 2001; Seltzer et al., 2003). Interestingly, however, improvements in the Comm domain for participants with ASD in the Shattuck et al.’s (2007) study were more modest than McDuffie et al. observed in their comorbid FXS and autism sample, particularly in non-verbal communication items, thereby again raising the possibility of different mechanisms underlying symptoms of autism in FXS and non-syndromic ASD. At the same time, however, it is important to recognize that there were also similarities between the two studies in symptoms that did not change. For example, limited friendships during adolescence characterized the non-syndromic ASD group in the Shattuck et al.’s (2007) study as well as both the FXS groups in the McDuffie et al. study; however, we cannot rule out the possibility that the friendship problems of the two groups may have different causes.

It is interesting to note that the autism symptom improvement observed in individuals with FXS by McDuffie et al. (2010) occurs at the same time as IQ declines reflecting a further slowing of cognitive development relative to typically developing peers (Kover et al., 2013). In the case of non-syndromic ASD, however, autism symptom improvement occurs in the context of stability in IQ (Baird, 2014; Magiati et al., 2014). Thus, these data provide further evidence that the mechanisms underlying at least some ASD symptoms are likely to be different in the two conditions. Again, reliance on a categorical ASD diagnosis would obscure those differences.

## SYMPTOM PROFILES IN FXS WITH AND WITHOUT COMORBID ASD

Numerous studies, including several we have conducted, have been designed to understand the symptoms and correlates distinguishing individuals with FXS who receive an ASD diagnosis from those with FXS who do not receive the diagnosis (see Budimirovic and Kaufmann, 2011 for a review). Although perhaps a more indirect approach than those considered in previous sections, these studies can provide insight into the essential impairment(s) distinguishing the two FXS subgroups and whether the difference is best explained as the result of the core impairments that define ASD in the non-syndromic case.

In the McDuffie et al.’s (2010) study described previously, ADI-R profiles were compared for 50 children and adolescents with FXS (both males and females) divided into those who met and did not meet criteria for autism. Comparisons were made for both the lifetime, or diagnostic, items (i.e., which described functioning largely at ages 4–5 years) and the items assessing current functioning. Importantly, McDuffie et al. controlled for the expected differences in IQ between the two groups, using non-verbal IQ (determined from the Leiter International Performance Scale) as a covariate in all comparisons.

Interestingly, a multivariate analysis of covariance did not yield a statistically significant difference between the groups for the RSI domain items for either the lifetime items or current age items. In contrast, statistically significant group differences associated with large effect sizes emerged for both the Comm domain items and the Restricted Interests and Repetitive Behaviors items, with the comorbid FXS–ASD group being more severely impaired. In interpreting these findings, McDuffie et al. argued that it is difficult to view the autism diagnosis in FXS as reflecting the same underlying problem as in non-syndromic autism in light of the finding that impairments in reciprocal social interaction are not what distinguishes individuals with comorbid FXS and ASD from their peers with FXS only, once IQ is controlled.

It should be noted that, in contrast to McDuffie et al. (2010), Hernandez et al. (2009) found that the groups with and without autism in their prospective longitudinal study of 3- to 8-year-olds with FXS were most discriminated by differences in adaptive socialization. IQ differences between the subgroups existed at two of three assessment points and were not controlled for in the Hernandez et al.’s (2009) study, complicating interpretation of their findings.

Rather than examining autism symptoms *per se*, several studies have focused on the correlates of the ASD diagnosis or ASD symptom severity within FXS. In one such study, McDuffie et al. (2012) were interested in determining the ways in which the profile of language impairments in FXS differed as a function of ASD diagnostic status and ASD symptom severity. The participants were 34 boys between the ages of 10 and 15 years, with a mean age near 13 years. The ADI-R and ADOS were administered, as were measures of receptive vocabulary (Peabody Picture Vocabulary Test—Third Edition, PPVT-3; Dunn and Dunn, 1997), receptive syntax (Test for Reception of Grammar—Second Edition, TROG-2; Bishop, 2003), expressive vocabulary (Expressive Vocabulary Test, EVT; Williams, 1997), and expressive syntax (Syntax Construction from the Comprehensive Assessment of Spoken Language, CASL; Carrow-Woolfolk, 1999). The sample was quite impaired, on average, with a mean non-verbal IQ (assessed via the Leiter International Performance Scale) near 45.

The first set of analyses in the McDuffie et al.’s (2012) study compared the language profiles of the 16 boys who met criteria for autism on both the ADI-R (using the original algorithm) and the ADOS (using the Gotham et al., 2007, revised diagnostic algorithm) and the 8 boys who did not meet criteria on either measure (*n* = 8), with ambiguous cases (i.e., those who met diagnostic criteria on only one measure) excluded. After controlling for group differences in non-verbal IQ and chronological age, no statistically significant differences emerged on any of the language measures. The lack of group differences in language is rather surprising in light of the especially serious receptive language impairments often reported for individuals with non-syndromic ASD (e.g., Kjelgaard and Tager-Flusberg, 2001; Ellis Weismer et al., 2010; Hudry et al., 2010; Kover et al., 2013), especially among boys with comorbid intellectual disability (Kover et al., 2014). It may be, however, that limited statistical power resulting from the small sample size led to the null findings, as the authors noted.

The second set of analyses included all 34 boys and examined the relationship between autism symptom severity and each of the language measures, controlling for age and non-verbal IQ. In this analysis, autism symptom severity was a significant predictor of both receptive vocabulary and receptive syntax, but not of either measure of expressive language skill. The finding of significant associations between autism symptom severity and receptive language is in keeping with expectations derived from what is known of non-syndromic ASD; that is, receptive language impairments are a core feature of the disorder and may be, in part, attributed to cumulative deficits in attending and responding to the referential cues of conversational partners (Mundy and Jarrold, 2010). In addition, several other studies have found individuals with comorbid FXS and autism to be especially impaired in receptive language relative to those with FXS alone (e.g., Rogers et al., 2001; Philofsky et al., 2004; Lewis et al., 2006; Hernandez et al., 2009).

Other differences have been found in the language profiles of individuals with FXS according to either autism diagnostic status or autism symptom severity. For example, autism symptom severity was negatively related to a measure of talkativeness in a conversational context in a sample of 10- to 17-year-old verbal males with FXS after controlling for non-verbal IQ (Kover et al., 2012); that is, individuals with more severe autism symptoms (assessed via the ADOS) were more verbally reticent, an association that would be consistent with the lack of motivation to engage in social interaction that is a hallmark of non-syndromic ASD (Chevallier et al., 2012).

Thus, as in the other types of data reviewed, the differences between individuals with FXS who do and do not meet criteria for ASD are not always consistent with expectations derived from the literature on non-syndromic ASD. In other words, a categorical ASD diagnosis in individuals with FXS masks nuances in the symptom profile that are of clinical significance. Particularly compelling empirical data in support of this point come from Budimirovic et al. (2006), who have shown that the ASD diagnosis in FXS appears to reflect the combination of both (a) social avoidance and (b) social indifference, each of which is a continuously distributed characteristic.

## NEUROBIOLOGICAL SUBSTRATES OF CORE ASD SYMPTOMS AND BEHAVIORAL CORRELATES IN FXS AND NON-SYNDROMIC CASES

There has been considerable research into the structural and functional characteristics of the FXS brain, as well as into other biomarkers of the pathology associated with the syndrome. Some of the data generated focus on differences that map onto autism symptoms in FXS and on differences between ASD in FXS and non-syndromic cases. If ASD is the same disorder in FXS and the non-syndromic case, it is reasonable to expect similar profiles across the two disorders in terms of biomarkers of pathology. Similarly, differences in biomarkers should correlate with ASD status or severity within FXS after other confounding factors are controlled. As is the case with behavioral data, evidence of both similarities and differences between the relevant subgroups has emerged, suggesting a deeper more nuanced picture than is captured by a simple categorical ASD diagnosis.

### BRAIN STRUCTURE AND FUNCTION

In terms of brain structure, Hoeft et al. (2011) used neuroimaging to study brain volumes in 1- to 4-year-olds with either FXS or non-syndromic ASD. These investigators found that both groups differed form typical controls, but in very different ways. In general, frontal and temporal regions thought to underlie social information processing were larger than controls for the non-syndromic ASD group but smaller in the FXS group. Moreover, there were no differences in brain volume according to ASD status with the group of participants with FXS. Similarly, Meguid et al. (2010) found that individuals with non-syndromic ASD had a thinner cortex in several brain regions relative to individuals with FXS and comorbid ASD. Other studies also have found structural differences between FXS and non-syndromic ASD, such as greater amygdala volume in non-syndromic ASD than in FXS, with the amygdala thought to be critical in emotional processing and responding (Hazlett et al., 2009). At the same time, however, there are data suggesting structural differences between individuals with FXS with and without a comorbid ASD diagnosis (e.g., Kaufmann et al., 2003).

In a study using functional magnetic resonance imaging (fMRI) to examine patterns of brain activation, we (Dalton et al., 2008) examined patterns of neural activation and concomitant eye gaze fixation during a task that involved in processing emotions conveyed via other people’s facial expressions. The participants were adolescents and adults with FXS or non-syndromic ASD, as well as typically developing controls. The two disordered groups each showed less activation relative to controls of the fusiform gyrus, a region integrally involved in face processing. Nevertheless, the participants with FXS showed increased activation of several other brain regions relative to both of the other groups, suggesting that emotion processing is approached somewhat differently by individuals with FXS. At the same time, however, autism symptom severity was correlated with activation in the fusiform gyrus, but not the amygdala (after controlling for IQ), in the group of participants with FXS. Unfortunately, the same metric of autism symptom severity was not available for the non-syndromic ASD group and thus, no analysis of within-group variation was conducted for that group.

### FMRP LEVELS

Fragile X mental retardation protein is the protein product that is reduced or absent in FXS. FMRP levels (measured in peripheral lymphocytes) have generally been found to be correlated with the severity of a wide range of behavioral symptoms in both males and females and throughout the life course. Several studies have examined the relationship between FMRP and autism status or symptom severity.

In the McDuffie et al.’s (2010) and Kover et al.’s (2013) studies already discussed, we examined the relationships among FMRP, IQ, and autism symptoms in adolescents with FXS. Although FMRP levels were significantly associated with symptoms in the domains of reciprocal social interaction, communication, and restricted interests and repetitive behaviors, none of the correlations remained significant after differences in non-verbal IQ were controlled. In the Kover et al.’s (2013) study, which was focused on examining the predictors of the age-related trajectory of IQ in adolescents with FXS, autism symptom severity was found to predict the intercept (i.e., level at the first assessment) of IQ; however, no prediction was afforded by autism symptom severity after FMRP levels were controlled, which also suggests that the FMRP-autism symptom severity is mediated by IQ. Other investigators also have found no difference in FMRP levels between individuals with FXS with and without an ASD diagnosis or other index of autism symptomatology once IQ is controlled (Cornish et al., 2004; Loesch et al., 2007).

In short, the FMRP findings do not generally validate at the biological level the distinction between those with and without comorbid ASD or between those with less and more severe ASD symptoms. Nevertheless, it is important to acknowledge that FMRP has been shown to be a central construct in the control of several important neural pathways implicated in a number of disorders, including schizophrenia (D’Antoni et al., 2014; Hamilton et al., 2014; Waltes et al., 2014), and non-syndromic ASD is likely to be included in that list as well.

## SUMMARY AND CONCLUSIONS

The picture emerging thus far from our data is one in which there are important differences in the behavioral manifestations, behavioral correlates, and neurobiological substrates of ASD in FXS relative to non-syndromic ASD. Moreover, these differences suggest that the underlying psychological and neurobiological problems may be partly different in the two conditions. In particular, we have found that:

1. Individuals with FXS who are diagnosed with ASD have less severe impairments in several of the social and communication symptoms that are diagnostic of ASD than do individuals with non-syndromic ASD, and these differences have emerged in a number of analyses and cohorts and across a variety of matching strategies. Even a hallmark symptom, such as pronoun reversal, distinguishes ASD in FXS and non-syndromic cases and is particularly interesting in light of the language and communication challenges experienced by males with FXS (Abbeduto et al., 2007). At the same time, however, there are areas of greater ASD symptom severity in FXS in the domain of restricted interests and repetitive behaviors.
2. Individuals with FXS who are diagnosed with ASD also present with a number of comorbid problems that further distinguish them from individuals with non-syndromic ASD. In particular, problems with anxiety and hyperactivity are more severe in FXS than in comorbid ASD. Moreover, there is some suggestive evidence that these problems may be driving the emergence of social interaction difficulties that over the course of development create a profile of impairments that lead to the ASD diagnosis in individuals with FXS. Although problems in the domains of anxiety and hyperactivity occur in ASD (Eussen et al., 2013; van Steensel et al., 2014), they do not appear to be causally linked to autism symptoms in the same manner in ASD as for FXS.
3. The developmental trajectory of ASD symptoms appears to differ between individuals with FXS and comorbid ASD and those with non-syndromic ASD. Although symptom abatement, at least during the late childhood and early adolescent years, occurs in both conditions, the improvements in communication appear more dramatic in FXS, although more prospective longitudinal data are needed to confirm this conclusion. The ASD symptom improvement during adolescence in FXS is occurring at the same time that cognitive development is actually slowing in FXS, a pattern not seen in non-syndromic ASD.
4. Within-syndrome comparisons of individuals with FXS with and without a diagnosis of autism have uncovered differences that are not always consistent with expectations for the “essence” of ASD. On the one hand, individuals with FXS and comorbid autism have more severe receptive language problems than do their FXS peers without an autism diagnosis. On the other hand, these two subgroups do not differ in terms of the social impairments that represent the core deficit of autism, once differences in IQ are controlled, although the data are not consistent across studies in the regard. Replication is thus required.
5. In terms of neurobiology, here too we see striking differences between FXS and nonsyndromic ASD in terms of brain structure as well as some more subtle differences in terms of brain function. At the same time, there are differences and similarities in brain structure as regards the distinction between individuals with FXS with and without comorbid ASD. Finally, FMRP does not appear to map strongly onto autism symptomatology in FXS.

Although these data strongly suggest that ASD is “not the same disorder” in the FXS and non-syndromic ASD populations, several caveats should be noted. First, in light of the clinical importance of a “mischaracterization” of the problem to be treated, the data we have presented are limited in many ways. Larger, more diverse, samples are needed to ensure that we understand the limits of generalizability of our findings. Examination of these issues across the entire life course is needed, and the samples studied must be enriched in terms of representation of females. Second, although the profile of relative impairments, relationships among impairments, and trajectories of impairment differ between individuals with FXS and comorbid ASD and individuals with non-syndromic ASD, there is considerable overlap, and not absolute difference. It is, however, premature to identify with any certainty the individual symptoms that are reliably common across the two disorders because of variations in sample characteristics and measures across studies and because a lack of differences between participant groups can often reflect limited statistical power. Nevertheless, the available data suggest that many communication impairments may be common across individuals with FXS and comorbid ASD and individuals with non-syndromic ASD (McDuffie et al., 2010, 2014; Losh et al., 2012), although not necessarily only those symptoms seen as diagnostic of ASD. Finally, the mechanisms, or psychological problems, underlying these differences between the two disorders are not fully explained by the data thus far, although anxiety (Kaufmann et al., 2008; Cordeiro et al., 2011), hyperactivity (Thurman et al., 2014), social withdrawal (Budimirovic et al., 2006; Kaufmann et al., 2008), and adaptive socialization (Budimirovic et al., 2006; Hernandez et al., 2009) may play central roles in the emergence of ASD in FXS. Ultimately, we believe that more studies of brain structure and function, as well as the biological levels intervening between the *FMR1* gene and behavior, are needed to tie these differences at the behavioral level to underlying pathology so that we can arrive at a level of explanation that can guide treatment.

We also note that part of the “confusion” about the comorbidity of FXS and ASD may be traced to reliance on the ADOS and the ADI-R. These measures work very well, and are the gold standards, for diagnosing ASD in non-syndromic individuals, which is the purpose for which they were designed. In the case of FXS (and perhaps other syndromes); however, the range of options for scoring might lead an impairment or unusual behavior to be scored as “autistic” because that is simply a better fit than a score reflecting unimpaired or typical. Perhaps a re-norming of these measures for FXS or the use of a coding scheme that allows finer distinctions to capture the difference between FXS and non-syndromic ASD impairments is needed.

The most important implication of the findings considered in this article is that the use of the categorical diagnosis of ASD in FXS masks important differences within the syndrome and between the syndrome and non-syndromic cases of ASD. These differences that are masked by the diagnosis include both individual symptoms that could be the targets of treatments and the underlying mechanisms that can inform the nature of the treatment required.

In conclusion, we still believe that the study of FXS can provide insights into non-syndromic ASD. As Harris (2011) argued, and our data support, those insights will come only when we abandon reliance on the categorical ASD diagnosis, and we move to the level of individual symptoms or constellations of symptoms that truly reflect the same underlying mechanisms in FXS and non-syndromic ASD. In the interim, it is naïve and counterproductive to assume that ASD is the same in FXS as it is in the non-syndromic case or that treatments will essentially “transfer” readily from one condition to the other.

### Conflict of Interest Statement

Leonard Abbeduto has received financial support to develop and implement behavioral outcome measures for fragile X syndrome clinical trials from F. Hoffman-LaRoche, Ltd, Roche TCRC, Inc., and Neuren Pharmaceuticals Limited. No other author has financial disclosures to make.

## ACKNOWLEDGMENTS

Preparation of this paper was supported by grants R01 HD054764, R01 HD024356, and U54 HD079125, all from the National Institute of Child Health and Human Development.
